# Angiogenesis and Melanoma

**DOI:** 10.3390/cancers2010114

**Published:** 2010-02-25

**Authors:** Domenico Ribatti, Tiziana Annese, Vito Longo

**Affiliations:** Department of Human Anatomy and Histology, University of Bari Medical School, Piazza G. Cesare, 11, Policlinico 70124, Bari, Italy; E-Mails: tiziana.annese@virgilio.it (T.A.); vito.vito@tiscali.it (V.L.)

**Keywords:** angiogenesis, antiangiogenesis, human melanoma, tumor progression

## Abstract

Angiogenesis occurs in pathological conditions, such as tumors, where a specific critical point in tumor progression is the transition from the avascular to the vascular phase. Tumor angiogenesis depends mainly on the release by neoplastic cells of growth factors specific for endothelial cells, which are able to stimulate the growth of the host’s blood vessels. This article summarizes the literature concerning the relationship between angiogenesis and human melanoma progression. The recent applications of antiangiogenic agents which interfere with melanoma progression are also described.

## 1. Introduction

Angiogenesis, *i.e.,* the formation of new vessels from pre-existing ones such as capillaries and post-capillary venules, plays a pivotal role during embryonal development and later, in adult life, in several physiological and pathological conditions, such as tumor and chronic inflammation, where angiogenesis itself may contribute to the progression of disease.

Angiogenesis is controlled by a balance between molecules that have positive and negative regulatory activity, and this concept has led to the notion of the angiogenic switch, which depends on an increased production of one or more positive regulators of angiogenesis [1]. Angiogenesis, and the production of angiogenic factors, are fundamental for tumor progression in the form of growth, invasion and metastasis, and practically all solid tumors growth occurs by means of an avascular phase followed by a vascular phase [2]. 

Human melanoma is produced by the transformation of an epidermidal melanocyte into a malignant cell and spreads in three ways: locally within the dermis; *via* the lymphatics, and *via* the bloodstream. The primary tumor grows horizontally through the epidermis. Over time, a vertical growth phase component develops in the primary tumor, and the melanoma begins to thicken and invade the lower levels of the dermis. Once a vertical growth phase has developed, metastasis becomes more likely, and there is a direct correlation between the thickness of the vertical growth phase component of a primary melanoma and the likelihood of metastasis [3]. In agreement with progression, melanoma acquires a rich vascular network [4,5], where an increasing proportion of tumor cells express the laminin receptor, which enables their adhesion to vascular wall [6]. A correlation between increased angiogenesis expressed as intratumoral microvessel density (MVD) and several parameters, such as poor prognosis, tumor thickness, overall survival and increased relapse rate, has been established in human melanoma [7,8,9,10,11]. The degree of angiogenesis in human melanoma depends on the concerted action of several angiogenic and antiangiogenic factors produced by various types of cells in the melanoma microenvironment; moreover, there is a strong relationship between inflammation, angiogenesis and metastasis in melanoma [12]. Multiple studies have examined the expression of pro-angiogenic growth factors and their receptors in melanoma. This review summarizes several aspects of melanoma angiogenesis and clinical implications.

## 2. Role of Classic Angiogenic Factors

Vascular endothelial growth factor (VEGF) is an angiogenic factor *in vitro* and *in vivo*, and a mitogen for endothelial cells with effects on vascular permeability [13]. The VEGF family includes VEGF-A, VEGF-B, VEGF-C, VEGF-D, VEGF-E and placental growth factor (PlGF). All the VEGF isoforms share common tyrosine kinase receptors [14]. VEGF-A bind with high-affinity to VEGFR-1 and VEGFR-2, and plays an essential role in angiogenesis: PlGF enhances angiogenesis by displacing VEGFR-1 only in pathological conditions and thereby making more VEGF available to bind VEGFR-2, by transmitting angiogenic signal through its receptor VEGFR-1 *via* a novel cross-talk; this causes activation of VEGFR-1 by PlGF which results in enhanced tyrosine phosphorylation of VEGFR-2 [15]. VEGF is expressed by tumor cells both *in vitro* and *in vivo*, increases vascular permeability and promotes the extravasation of plasma proteins and other circulating macromolecules from tumor vessels [13].

Melanoma cells produce and secrete VEGF-A [16,17]. Inoculation of human melanoma cells transfected with VEGF-A into immunodeficient mice results in an increase of vascularization and microvessel permeability of melanoma xenografts [18,19,20,21].

Ribatti *et al.* [22] demonstrated that in human primary melanoma an increased microvascular density, a strong VEGF-A immunoreactivity of tumor cells, an increased vessel diameter and an high number of connections of intraluminal tissue folds with the opposite vascular wall; expression of intussusceptive angiogenesis, are correlated to an higher tumor thickness.

Yu *et al*. [23] demonstrated that in human melanoma xenografts, overexpression of VEGF_121 _and VEGF_165 _was responsible for tumor growth, whereas overexpression of VEGF_189 _did not induce tumor growth.

The transition from horizontal to vertical growth phase in human primary melanoma and from primary to metastatic melanoma was associated with an increased VEGF-A expression and accumulation in the tumor stroma [24,25,26]. Claffely *et al*. [27] subcutaneously implanted a melanoma cell line overexpressing VEGF-A and demonstrated a vasoproliferative response, while Kusters *et al*. [28] reported that VEGF-A caused vasodilatation and increase of vascular permeability in a mouse brain metastasis model of human melanoma. 

Marcellini *et al*. [29] used transgenic mice overexpressing PlGF in the skin under the control of the keratin 14 promoter, which showed a hypervascularized phenotype of the skin and increased levels of circulating PlGF with respect to their wild-type littermates. Transgenic mice and controls were inoculated intradermally with B16-BL6 melanoma cells. The tumor growth rate was five-fold increased in transgenic animals compared to wild-type mice and tumor vessel area was increased in transgenic mice as compared to controls. Moreover, the number and size of pulmonary metastases were significantly higher in transgenic mice compared to wild-type mice and PlGF-promoted tumor cell invasion of the extracellular matrix. 

Ugurel *et al*. [30] showed that VEGF-A, FGF-2 and IL-8 were strongly correlated with poor clinical outcome and were independent predictive factors for overall survival in melanoma patients. Pelletier *et al*. [31] reported that in patients with stage I-II-III primary melanoma, the absence of an increase in plasma VEGF-A levels during follow-up was associated with remission with a predictive value of 90%. Sabatino *et al*. [32] reported that high serum levels of VEGF-A and fibronectin correlated with lack of clinical response to high-dose treatment with IL-2. Both an increase [31] or a decrease [33,34] of VEGF serum levels after treatment has been described.

Lymphangiogenesis is an important step in tumor progression. VEGF-C has been characterized as a lymphangiogenic growth factor signaling *via* VEGFR-2 and VEGFR-3. VEGF-C has been detected on endothelial and tumor cells [35] and mediates tumor lymphangiogenesis and invasion of the neoplastic cells into lymphatic vessels. VEGF-C overexpressing tumors increase intratumoral lymphangiogenesis by activating the VEGF-C/VEGFR-3 axis in lymphatic endothelial cells, enhancing metastatic spread *via* the lymphatic and peritumoral amounts of lymphatic vessels [36]. VEGF-A also acts as a lymphangiogenic factor and tumor-derived VEGF-A promotes expansion of the lymphatic network within draining, sentinel lymph nodes, even before these tumors metastasize [37].

VEGF-C was found to be expressed in primary cutaneous melanomas [38]. Melanomas overexpressing VEGF-C have increased intratumoral blood and lymph vessels [39] and a significant increase in intratumoral lymphatics was observed in metastatic primary melanomas [40]. Moreover, lymphangiogenesis and metastasis was increased in sentinel lymph nodes in carcinogenesis experiments in transgenic mice overexpressing VEGF-C in the epidermis [41]. Once the metastatic cells arrived at the sentinel lymph nodes, the extent of lymphangiogenesis at these sites increased. In mice with metastasis-containing sentinel lymph nodes, tumors that expressed VEGF-C were more likely to metastasize to additional organs, such as distal lymph nodes and lungs, while no metastases were observed in distant organs in the absence of lymph node metastases [41]. Mouawad *et al*. [42] demonstrated that pre-treatment serum VEGFR-3 levels significantly correlate to chemoresistance and poor prognosis in metastatic melanoma patients. 

Fibroblast growth factor-2 (FGF-2) is one of the best characterized and investigated pro-angiogenic cytokines and a large body of research has implicated FGF/FGFreceptors (FGFRs) as having a role in tumorigenesis [43]. Numerous studies have attempted to establish a correlation between intratumoral levels of FGF-2 mRNA or protein and intratumoral microvascular density in cancer patients [43].

Reed *et al*. [44] demonstrated that invasive human melanoma and metastatic melanoma expressed FGF-2 mRNA, whereas melanoma *in situ* and benign melanocytic nevi did not. Moreover, a significant relationship between high microvascular density and expression of FGFR_4_ has been described [45]. Antisense targeting of FGF-2 in melanoma cells completely blocked tumor growth and inhibited tumor angiogenesis *in vivo* [46]. Finally, Tsunada *et al*. [47] demonstrated a VEGF-dependent neovascularization in a mouse melanoma model induced by FGF-2.

Kurschat *et al*. [48] demonstrated that endostatin and FGF-2 are useful as a diagnostic markers for early detection during disease progression. Low IL-8 and FGF-2 concentrations at the beginning of interferon-alfa 2 beta (IFN-α 2b) adjuvant treatment have been shown to be associated with longer recurrence free-survival [49]. 

The angiopoietin (Ang) family comprises at least four secreted proteins, Ang-1, Ang-2, Ang-3 and Ang-4, all of which bind to the endothelial-specific receptor tyrosine kinase Tie-2. It is well documented that Angs play a critical role in endothelial sprouting, vessel wall remodeling and pericyte recruitment [50].

Helfrich *et al*. [51] demonstrated that Ang-2 acts as an autocrine regulator of melanoma cell migration and invasion, is expressed by tumor-associated endothelial cells and circulating levels of Ang-2 correlate with tumor progression and overall survival in melanoma patients. Interference with the Tie-2 pathway results in a significant inhibition of angiogenesis in melanoma [52,53,54,55]. 

Studies on targeted knock-out mice have provided evidence of an essential role for transforming growth factor beta (TGF-β) signaling in the formation of the vascular system [56]. TGF-β promotes melanoma angiogenesis by stimulating the expression of VEGF [57]. 

## 3. Role of Molecules and Cells Involved in Inflammation and Thrombosis

Interleukin-8 (IL-8) signaling promotes angiogenic responses in endothelial cells, increases proliferation and survival of endothelial and cancer cells, and potentiates the migration of cancer cells, endothelial cells, and infiltrating inflammatory cells at the tumor site. Accordingly, IL-8 expression correlates with the angiogenesis, tumorigenicity, and metastasis of tumors in numerous xenograft and orthotopic *in vivo* models. An increased expression of IL-8 and its receptors CXCR1 and CXCR2 has been demonstrated in cancer cells, infiltrating neutrophils, tumor-associated macrophages and endothelial cells, suggesting a function as a regulatory factor within the tumor microenvironment [58]. 

Primary and metastatic melanoma cells constitutively secrete IL-8, whereas non-metastatic cells produce low to negligible levels of IL-8 [59,60]. Transforming growth factor beta-1 (TGF-β_1_)selectively induces IL-8 expression in highly metastatic A375SM melanoma cells, but not in A375P non-metastatic parental cells [61]. Overexpression of IL-8 and its receptors parallel tumor progression, metastatic potential and angiogenesis in human melanoma [62,63,64], and neutralizing antibodies against IL-8 receptors inhibit melanoma angiogenesis [63,65]. Singh *et al*. [66] generated mCXCR2 (−/−), mCXCR2 (+/−), and wild-type nude mice following a cross between BALB/c mice heterozygous for nude (+/−) and heterozygous for mCXCR2 (+/−), and demonstrated a significant lower number of microvessels in tumors from mCXCR2 (−/−) and mCXCR2 (+/−) mice as compared with tumors from wild-type mice. Rofstad and Halsor [67] demonstrated a correlation between hypoxia, IL-8, angiogenesis and metastasis in human melanoma xenografts. Moreover, neutralizing antibodies against IL-8 reduced the vascular density and the incidence of metastases.

A significant correlation between IL-8 serum concentration and tumor load has been shown [68]. Brennecke *et al*. [69] showed that low IL-8 serum levels after chemotherapy correlated to clinical response in stage IV melanoma patients, whereas elevated serum levels of VEGF and FGF-2 persisted following the initial cytostatic administration.

Platelet-activating factor (PAF) is a potent proinflammatory phospholipid with diverse pathological and physiological effects. It mediates processes as diverse as wound healing, physiological inflammation, apoptosis, angiogenesis, and reproduction Moreover, cancer cells and activated endothelial cells expose PAF-receptor on their membrane surface. PAF binding to its receptor induces several pathways that result in the onset and development of tumor-induced angiogenesis and metastasis [70].

PAF and its receptor act as important modulators of melanoma angiogenesis. Protease activated receptor-1 (PAR-1) is overexpressed in highly metastatic melanoma cell lines and in metastatic lesions of melanoma patients [71,72]. The activation of PAR-1 is directly responsible for the expression of genes involved in melanoma angiogenesis, such as IL-8, VEGF and platelet derived growth factor (PDGF) [73].

Yin *et al*. [74] characterized a stable PAR-1 metastatic melanoma cell line (C113) and detected an higher expression of VEGF in C113 cells than in non-metastatic parental cells. In addition, they injected subcutaneously into mice C113 cells mixed with Matrigel and demonstrated an higher number of blood vessels in plugs containing C113 cells than in those containing non-transfected cells.

Villares *et al*. [75] used systemic delivery of PAR-1 small interfering RNA (siRNA) incorporated into neutral liposomes to inhibit melanoma growth *in vivo* and found a concomitant decrease in VEGF, IL-8, matrix metalloproteinase-2 (MMP-2) expression levels, as well as decrease in blood vessels density in tumor samples from PAR-1 siRNA treated mice compared to control animals.

Biancone *et al*. [76] reported that inhibition of PAF activity in B16 melanoma cells led to a significant decrease in tumor vascularization and growth. Ko *et al*. [77] demonstrated that a single intraperitoneal injection of PAF induced an enhanced lung metastatic potential of melanoma B16 cells through an increase of MMP-9 expression in blood vessels. Melnikova *et al*. [78] demonstrated that the PAR-1-mediated expression of melanoma adhesion molecule MCAM/MUC18, a critical marker of melanoma metastasis, is mediated by the activation of the PAF receptor.

The platelet-derived growth factor (PDGF) family comprises four family members (PDGF-A to PDGF-D), which bind, with distinct selectively, to receptor tyrosine kinases PDGFR-A and PDGFR-B expressed on endothelial cells and smooth muscle cells [79]. Moreover, PDGF play a critical role in pericyte recruitment in both normal and tumor vessels [80]. 

Robinson *et al*. [81] demonstrated that human melanoma xenografts derived from B16 cells transfected with PDGF-BB show vessels with an higher pericyte coverage as compared to control cells. Moreover, they demonstrated by MRI analysis that PDGF-BB induced a decrease of vessel caliber and an increased degree of perfusion of tumor blood vessels. Suzuki *et al*. [82] demonstrated that the total vessel area and the average vessel surface were higher in tumors grown in mice carrying an activated PDGF receptor beta injected with B16 melanoma cells as compared to wild-type mice. Finally, Faraone *et al*. [83] demonstrated that PDGFR-A strongly inhibits melanoma growth *in vitro* and *in vivo* and that melanoma cells overexpressing PDGFR-A give rise to tumors markedly smaller in weight and with strongly reduced tumor angiogenesis compared with controls. These findings may suggest new therapeutic approaches effective at clinical kevel by using inhibitors of PDGFRs.

There is increasing evidence to support the view that angiogenesis and inflammation are mutually dependent. During inflammatory reactions, immune cells synthesize and secrete pro-angiogenic factors that promote neovascularization. On the other hand, the newly formed vascular supply contributes to the perpetuation of inflammation by promoting the migration of inflammatory cells to the site of inflammation [84]. 

An increase in mast cell density has been described in invasive melanoma as compared to benign nevi and *in situ* melanoma [85]. Ribatti *et al*. [86] demonstrated an high correlation between microvessels count, tumor cells reactive to FGF-2, mast cells count and tumor progression in human melanoma. Guidolin *et al*. [87] reported that the spatial distribution of mast cells in melanoma was characterized by a close spatial association between mast cells and vessels. Tóth-Jakatics *et al*. [86,88] demonstrated that in cutaneous malignant melanomas intradermal mast cells are immunoreactive to VEGF and demonstrated a prognostic significance of mast cell density and microvascular density in melanoma patients, showing a shorter survival rate in patients with highly values of these parameters. 

Macrophage infiltration correlates with tumor stage and angiogenesis in malignant melanoma [89,90]. Melanoma cells secrete monocyte chemotactic protein-1 (MCP-1) and CC chemokine ligand-5 (CCL5), a powerful activator of monocytes/macrophages, dendritic cells and mast cells [91]. Tumor derived MCP-1 and CCL5 induce macrophages to secrete angiogenic factors, such as IL-8, VEGF, MMP-9, FGF-2, tumor necrosis factor alfa (TNF-α) and PDGF [92]. In turn, TNF-α secreted by macrophages increases the secretion of VEGF and IL-8 from melanoma cells [89]. Varney *et al*. [93] demonstrated that macrophage conditioned medium significantly up-regulated IL-8 expression in human malignant melanoma *in vitro*. Furthermore, they demonstrated that co-culture of melanoma cells with monocytes enhanced VEGF-A secretion, and monocyte conditioned medium enhanced melanoma cell expression of VEGF-A [94].

## 4. Role of Non-Classic Angiogenic Factors

Melanotransferrin (MTf), the membrane-bound human melanoma antigen p97, binds to plasminogen and stimulates its activation, thus regulating a crucial step involved in angiogenesis.

MTf is highly expressed in melanoma cells as compared to normal melanocytes, and plays a critical role in melanoma cell proliferation and tumorigenesis [95]. Sala *et al*. [96] reported that MTf induced chemotactic migration of vascular endothelial cells in a Boyden chamber and angiogenesis *in vivo* in the chick embryo chorioallantoic membrane (CAM) assay. Moreover, a soluble form of MTt inhibited angiogenesis *in vivo* [97].

Angiotensin II (Ang II) is angiogenic *in vivo* in the CAM and in the rabbit cornea assay [98,99] and stimulates the growth of quiescent endothelial cells *via* angiotensin II type 1 receptors (AT_1_Rs) [100].

Human melanomas expressing both Ang II and AT_1_Rs and a significantly reduction of capillary density was found in melanoma of AT_1_R deficient mice [101]. Otake *et al*. [102] demonstrated that Losartan, an antagonist of AT_1_R, inhibited tumor growth in murine melanoma. 

Endothelins (ETs) are a family of hypertensive peptides, mainly secreted by endothelial cells and overexpression of ET-1 and its receptors has been found in tumors [103].

Endothelin B-receptor (ET_B_-R) is overexpressed in human melanoma, activation of the ET_B_-R pathway increases the expression of MMP-2 and MMP-9 and ET_B_-R antagonist induced an inhibition of tumor growth and a decrease of vascular density [104]. Moreover, ET-1 and ET-3 promote invasive behaviour *via* hypoxia inducible factor 1 alpha (HIF-1α) in human melanoma cells [105].

## 5. Role of Endogenous Inhibitors of Angiogenesis

Thrombospondin-1 (TSP-1) was the first protein to be recognized as a naturally occurring inhibitor of angiogenesis by Bouck and collaborators in their search for proteins upregulated by tumor suppressor genes [106].

Rofstad *et al*. [107] reported that melanoma angiogenesis, lung colonization and spontaneous pulmonary metastasis were inhibited in mice overexpressing TSP-1. Furthermore, Rofstad *et al*. [108] demonstrated that TSP-1 treatment prevents growth of dormant lung micrometastasis after surgical resection and curative radiation therapy of the primary tumor in human melanoma xenografts. 

Angiostatin was discovered in 1994 by M. O’Reilly in the Folkman laboratory based on Folkman’s hypothesis that a primary tumor could suppress its remote metastasis because expression of proangiogenic proteins within the primary tumor exceed the generation of antiangiogenic proteins resulting in the vascularization and growth of the primary tumor. Angiostatin specifically inhibited the proliferation of growing vascular endothelial cells and the growth of primary tumors by up to 98% and was able to induce regression of large tumors and maintain them at a microscopic dormant size [106]. In 1997, O’Reilly isolated and purified another angiogenesis inhibitor from a murine hemangioendothelioma called endostatin. Endostatin counteracts virtually all the angiogenic genes upregulated by either VEGF or FGF-2 and also downregulates endothelial cell Jun B, HIF-1α, neuropilin and the epidermal growth factor receptor. However, clinical trials using endostatin in cancer patients have yielded only sporadically positive results [106].

In a mice model of leptomeningeal melanoma, treatment with angiostatin resulted in prolonged survival [109]. Yang *et al*. [110,111] reported that in mouse model of uveal melanoma, treatment with angiostatin at low doses resulted in decreased hepatic micrometastasis associated with a reduction of VEGF expression. Kim *et al*. [112,113] have demonstrated that transfection of angiostatin and endostatin resulted in an inhibition of neovascularization and tumor progression in B16 mouse melanoma tumors. Moreover, in a murine melanoma brain metastatic model, melanoma expressing endostatin displayed a reduced capacity to recruit an adequate vascular micrometastases supply [114] and injection of endostatin-recombinant plasmid into B16 melanoma in C57BL/6J mice followed by local x-irradiation inhibited tumor growth with a marked decrease of intratumoral vascularization [115].

IL-12 and IL-18 are IFNγ inducing cytokines with an antiangiogenic activity. Both treatment with IL-12 of mice with tumors and increased IL-12 delivery through gene transfer resulted in decreased tumor growth [106].

Airoldi *et al*. [116] transplanted the IL-12 receptor beta 2 gene and B16 melanoma cells into syngenic mice, which displayed higher endogenous serum levels of IL-12 and developed smaller B16 tumors than wilde-type mice. These tumors showed reduced microvascular density and defective microvessels. Heinzerling *et al*. [117] injected plasmid DNA encoding human IL-12 into lesions of patients with stage IV melanoma and demonstrated in two patients a stable disease and in one complete remission. Finally, Shimizu *et al*. [118] have demonstrated, using B16 melanoma cells in a murine model, that IL-27 exerted antiangiogenic and antitumor activities.

## 6. Antiangiogenic Therapy

Numerous clinical trials in patients with advanced metastatic melanoma indicate that melanoma is highly resistant to conventional cytotoxic chemotherapy and immunotherapy. Therapeutic options for metastatic melanoma are very limited, mainly palliative, and show response in only approximately 20% of all cases. Various experimental approaches have been conducted to evaluate the efficacy of an antiangiogenic molecules in melanoma treatment.

Inhibition of VEGF activity *via* neutralizing antibodies, VEGF antisense, RNA interference, oral VEGF receptor inhibitors, and anti-VEGF receptor vaccines are all effective strategies to slow the growth and metastasis of human melanoma [119,120,121,122,123,124]. The anti-VEGF antibody bevacizumab has Food and Drug Administration approval for certain types of breast cancer, non-small cell lung cancer, and metastatic colo-rectal cancer. Multiple phase I or II clinical trials are being conducted with bevacizumab alone or in combination in patients with metastatic melanoma. The administration of bevacizumab as a single agent did not reduce the tumor burden of patients with metastatic melanoma, but induced a prolonged disease stabilization (24 to 146 weeks) in a subset (8/32) of patients, including five patients treated with bevacizumab alone and three treated wuth bevacizuman plus interferon α2b [125]. Perez *et al*. [126] reported in a phase II trial that the combination of carboplatin, paclitaxel and bevacizumab resulted in a more efficacy response. Of the 53 patients enrolled, nine (17%) achieved partial remission, and another 30 (57%) achieved stable disease for at least eight weeks. Median progression-free survival and medial overall survival were 6 and 12 months, respectively.

Thalidomide inhibits vasculogenic mimicry channel and mosaic vessels formation in melanoma [127]. Hwu *et al*. [128] reported a 32% of objective tumor response and acceptable toxicity in a phase II trial with temozolamide and thalidomide in metastatic melanoma patients. A significant response was also found in patients with brain metastatic melanoma [129]. On the contrary, a very low response rate was found in patients with metastatic melanoma treated with combination between thalidomide, temozolamide and whole brain irradiation [130]. Ott *et al*. [131] reported that the combination of dacarbazine and thalidomide demonstrated low efficacy and unacceptable toxicity. Thalidomide in combination with IFNα-2b demonstrated a lack of response and was associated with multiple severe toxicities [132]. 

Glaspy *et al*. [133] evaluated lenalidomide in metastatic melanoma patients previously treated with chemotherapy and showed an overall response rate at higher dose of only 5.5%. Finally, sorafenib, a multi-kinase inhibitor, was ineffective against melanoma as a single agent [134] and the addition of sorafenib to chemotherapy did not improve the response rate [135]. 

It is critical to take potential adverse effects, such as the high rate of severe thromboembolic events, into account when antiangiogenic molecules have used in the treatment of melanoma alone or in combination with other medications.

## 7. Concluding Remarks

Several clinical studies are currently being conducted to assess the effects of angiogenesis inhibitors in the treatment of patients with metastatic melanoma. A therapeutic approach that combines angiogenesis inhibitors with cytotoxic agents seems more likely to result in a clinical benefit for patients than antiangiogenic treatment alone.

Most regimens combining cytotoxic agents with antiangiogenic molecules simply administer the agents at the same time, with no attention to scheduling and timing of treatment. Is seems that antiangiogenic therapy may be more beneficial if given before the administration of chemotherapy.

It is important to note that recent reports suggest that antiangiogenic therapy actively promotes tumor invasion and metastasis [136,137]. Increased invasiveness might result from enhanced expression of various cytokines induced by the treatment or from hypoxia-driven effects, including transcriptional activation of the hepatocyte growth factor receptor c-Met. Studies in VEGF-A null tumor cells in the RIP-Tag model suggest that loss of VEGF signaling in tumor cells stimulated local invasion even if the overall effects were beneficial because the loss of VEGF in tumor cells reduced tumor growth and prolonged survival [137]. Thus, combined modality treatment with antiangiogenic and antiinvasive therapies might exert beneficial therapeutic effects.
